# A Comprehensive Review of the Current State of Robot-assisted Laparoscopic Salvage Prostatectomy

**DOI:** 10.1590/S1677-5538.IBJU.2024.0126

**Published:** 2024-04-30

**Authors:** Parth U. Thakker, Maxwell Sandberg, Ashok K. Hemal, Alejandro R. Rodriguez

**Affiliations:** 1 Department of Urology Atrium Health Wake Forest Baptist Medical Center Winston-Salem NC USA Department of Urology, Atrium Health Wake Forest Baptist Medical Center, Winston-Salem, NC, USA

**Keywords:** Prostatic Neoplasms, Urologic Surgical Procedures, Prostatectomy

## Abstract

**Background and Objective:**

Salvage robot assisted radical prostatectomy (sRARP) is performed for patients with biochemical or biopsy proven, localized prostate cancer recurrences after radiation or ablative therapies. Traditionally, sRARP has been avoided by lower volume surgeons due to technical demand and high complication rates. Post-radiation sRARP outcomes studies exist but remain few in number. With increasing use of whole gland and focal ablative therapies, updates on sRARP in this setting are needed. The aim of this narrative review is to provide an overview of recently reviewed studies on the oncologic outcomes, functional outcomes, and complications after post-radiation and post-ablative sRARP. Tips and tricks are provided to guide surgeons who may perform sRARP.

**Materials and Methods:**

We performed a non-systematic literature search of PubMed and MEDLINE for the most relevant articles pertaining to the outlined topics from 2010-2022 without limitation on study design. Only case reports, editorial comments, letters, and manuscripts in non-English languages were excluded.

**Key Content and Findings:**

Salvage robotic radical prostatectomy is performed in cases of biochemical recurrence after radiation or ablative therapies. Oncologic outcomes after sRARP are worse compared to primary surgery (pRARP) though improvements have been made with the robotic approach when compared to open salvage prostatectomy. Higher pre-sRARP PSA levels and more advanced pathologic stage portend worse oncologic outcomes. Patients meeting low-risk, EAU-biochemical recurrence criteria have improved oncologic outcomes compared to those with high-risk BCR. While complication rates in sRARP are higher compared to pRARP, Retzius sparing approaches may reduce complication rates, particularly rectal injuries. In comparison to the traditional open approach, sRARP is associated with a lower rate of bladder neck contracture. In terms of functional outcomes, potency rates after sRARP are poor and continence rates are low, though Retzius sparing approaches demonstrate acceptable recovery of urinary continence by 1 year, post-operatively.

**Conclusions:**

Advances in the robotic platform and improvement in robotic experience have resulted in acceptable complication rates after sRARP. However, oncologic and functional outcomes after sRARP in both the post-radiation and post-ablation settings are worse compared to pRARP. Thus, when engaging in shared decision making with patients regarding the initial management of localized prostate cancer, patients should be educated regarding oncologic and functional outcomes and complications in the case of biochemically recurrent prostate cancer that may require sRARP.

## INTRODUCTION

In 2023, almost 288,000 new cases of prostate cancer were diagnosed in the US alone (1). Standard treatment options include watchful waiting, active surveillance, radiation therapy, and radical prostatectomy. Radiation therapy and primary robot-assisted laparoscopic radical prostatectomy (pRARP) both represent definitive treatment modalities for localized prostate cancer. Rates of post-radiation biochemical recurrence (BCR) range from 20-50% at 10 years while 30-50% of men with localized prostate cancer treated with radiation experience biochemical relapse (2). BCR prostate cancer after pRARP is most commonly managed with radiation therapy (3). Radiotherapy given in the salvage setting is associated with higher acute and late genitourinary and gastrointestinal toxicities compared to primary radiotherapy (4). Conversely, post-radiotherapy BCR is traditionally managed with salvage RARP (sRARP). While complication rates after salvage open prostatectomy were higher than primary open prostatectomy (5-8), complication rates after sRARP were near those of pRARP (9, 10). Nonetheless, sRARP is generally performed at high volume centers.

Currently, EAU guidelines recommended sRARP as a management option for those with post-radiotherapy BCR, as these men experienced a significant benefit in all survival outcomes compared to those with more poor disease features (11). Unfortunately, many of these studies report short-term outcomes after sRARP. Additionally, many new therapeutic options for the management of localized prostate cancer have gained popularity including cryoablation, high-intensity focused ultrasound (HIFU), irreversible electroporation (IRE), and transurethral ultrasound ablation (TULSA). While focal and whole-gland ablative therapies have emerged as integral components in the armamentarium of urologists, the landscape of managing prostate cancer recurrences after ablative therapies remains relatively uncharted. This narrative review seeks to summarize the oncologic and functional outcomes, complications, and technical details of sRARP performed for biochemically recurrent prostate cancer after radiation and ablative therapies. Details regarding the surgical approach to these patients is provided.

## MATERIALS AND METHODS

We performed a non-systematic literature search of PubMed and MEDLINE in November of 2023 to identify and select manuscripts from 2010-2022. The search key words included “robotic salvage prostatectomy” and “primary radical prostatectomy” in combination with “radiation therapy”, “brachytherapy”, “androgen deprivation”, “proton beam”, “cryoablation”, “high-intensity focused ultrasound”, “transurethral ultrasound ablation”. We manually reviewed all resulting manuscripts relevant to the topic. We also reviewed the references lists of review articles to include other papers relevant to the topic. Case reports, editorials, letters to the editor, and articles in non-English language were excluded.

## RESULTS

### Radiorecurrent Salvage Prostatectomy

Approximately 1/3 of patients with localized prostate cancer undergo primary non-surgical management (12). Biochemical recurrence after radiation is defined by the American Society for Therapeutic Radiation and Oncology (ASTRO) as a rise in serum PSA by ≥2 ng/mL from nadir levels (13). Up to 60% of patients are thought to experience BCR at 10 years [3]. Treatment of this patient population is critical as they are at increased risk of developing metastatic disease and prostate cancer specific mortality (14). The management options for radiorecurrent salvage prostatectomy are limited. sRARP has traditionally been associated with a higher complication rate compared to primary radical prostatectomy (15, 16). As such, salvage radical therapy is typically offered to those with > 10-year life expectancy, no evidence of metastatic disease, and those willing to accept the potential for higher complication rates and less favorable functional outcomes. Recently the European Urologic Association (EAU) has created a risk stratification for both post-radiation and post-prostatectomy BCR, which has been validated (17). Low risk, post-radiation EAU-BCR is defined as time to BCR > 18 months and biopsy Gleason grade < 4. High risk, post-radiation EAU-BCR is defined as time to BCR < 18 months and biopsy Gleason grade ≥ 4. These criteria may also be applied to patients undergoing focal ablation.

### S-RARP AFTER RADIATION THERAPY

#### a.Outcomes of Radiotherapy for localized prostate cancer

Radiotherapy for prostate cancer has evolved in respect to dosing, duration, fractionation, and intensity. The use of androgen deprivation therapy has also evolved and become an adjunctive treatment for many patients with advanced prostate cancer. The best measure of successful radiation therapy is debated and may be related to BCR, cancer-specific survival (CSS), metastasis-free survival (MFS), or overall survival (OS). The primary literature and meta-analyses have reported conflicting results relative to the benefit of any given treatment modality. Generally, radiation therapy is a safe and efficacious treatment option for men with localized prostate cancer. Herr et al. found a 98%, 97% and 90% CSS, a 96%, 92%, and 80% MFS, and a 77%, 71%, and 62% OS, for low-, intermediate-, and high-risk prostate cancer at a median follow up of 8.7 years (18). A SEER database study by Guo et al. found a cancer specific mortality rate of 2.3%, 2.0%, and 1.8% in heptogenarian patients with low- or intermediate-risk disease receiving external beam radiation therapy (EBRT), brachytherapy, or combination therapy, respectively (19). When compared to pRARP for localized prostate cancer, radiation therapy has been associated with similar rates of BCR, CSS, MFS, and OS (20). In a meta-analysis of patients undergoing pRARP or EBRT for all risk prostate cancer, both OS and CSS were better for patients undergoing pRARP and BCR free survival was better for patients undergoing EBRT at 10 years (21). This was further demonstrated by 15-year follow-up of men with prostate cancer in which similar rates of cancer specific mortality (2.2% vs 2.9%) and rate of metastasis (4.7% vs 5.9%) were found regardless of pRARP or radiotherapy, respectively (22). While outcomes of EBRT appear to be excellent, BCR after radiotherapy is associated with decreased survival compared to post-pRARP BCR (23). Thus, an investigation of the literature to determine the oncologic outcomes and complications after post-radiation, sRARP, is critical.

#### b.Oncologic Outcomes of open and robotic salvage radical prostatectomy after Primary Radiotherapy

Primary radiotherapy provides a noninvasive and effective treatment option for localized prostate cancer, however the optimal salvage therapy for radio-recurrent prostate cancer has not been determined. Furthermore, there remains a paucity of evidence to favor one approach. EBRT, brachytherapy, thermal ablation, androgen deprivation therapy, open and sRARP are all used in the salvage setting however, large scale, comparative, studies and randomized trials are scarce. Relevant studies vary widely in terms of primary outcomes measured and follow-up duration. While BCR-free survival is consistently reported, PFS, MFS, CSS, and OS are reported inconsistently, and varying definitions have been reported with the most critical difference being the PSA cutoff for defining BCR.

A majority of available literature pertains to salvage prostatectomy utilizing the open approach. Survival parameters for these can be found in Tables 1 and 2 with very few studies providing survival characteristics at 10 years. Among men undergoing open salvage prostatectomy, 5- and 10-year BFR free survival ranged from 39-61% and 31-48%, 5- and 10-year MFS ranged from 75-90% and 65-77%, 5- and 10-year CSS ranged from 89-95% and 65-83%, and OS ranged from 84-95% and 52-77%, respectively. As in the primary setting, curative intent is critical in the salvage setting in patients who have a reasonable life expectancy and as such understanding the variables that may impact survival statistics is critical. Exemplified in a study by Gheiler et al. 40 patients undergoing sRARP, non-organ confined disease, seminal vesicle invasion, and lymph node involvement were all negative prognostic factors for disease-free survival (24). Pre-sRARP PSA >10 ng/mL was found to be associated with a worse BCR-free survival, though not statistically different (73.7% vs 31.6%, p=0.65) (24). Similar findings were reported by Lerner et al. as patients with a pre-sRARP PSA > 10ng/mL had a lower PFS though not meeting statistical significance levels (70% vs 47%, p= 0.057) (25). Rodgers et al. found a PSA value >10ng/mL in the pre-sRARP setting to be the only predictive factor for lower PFS which was corroborated by Ward et al. in their study of 138 patients undergoing post-radiotherapy sRARP (26, 27). In one of the larger studies of 404 patients, Chade et al. reported a 10-year BCR free survival of 37% and found that higher pre-sRARP PSA levels and higher pathologic Gleason score were associated with higher rates of disease progression and metastasis (15).

**Table 1:** sRARP after radiation therapy failure. Studies in the narrative review which reported outcomes specifically for radiation therapy failure or in whom the majority of the cohort was radiation failure are included. Study type is reported as either retrospective (R) or retrospective comparative (RC).

Study	Therapy	N	Study type	Median/mean follow-up time (years)	Nerve sparing (unilateral or bilateral), n (%)	Median/Mean operative time (minutes)	Postoperative complications, n (%)	Positive surgical margin, n (%)	Upstaging on sRARP specimen, n (%)	BCR, n (%)	Continence (1-year), n (%)	Continence (>1 year), n (%)	Potency, n (%)
Gheiler et al. (24)	Radiation	30	R	3.1	-	222	10 (33)	6 (20)	-	15 (50)	-	15 (50)	-
Lerner et al. (25)	Radiation	79	R	5.3	-	-	35 (44)	24 (30)	-	19 (24)	28 (35)	-	-
Rogers et al. (26)	Radiation	39	R	3.3	-	264	25 (64)	15 (38)	21 (54)	29 (72)	-	18 (58)	-
Ward et al. (27)	Radiation	138	R	3.1	-	-	55 (40)	31 (22)	-	72 (52)	52 (40)	-	-
Chade et al. (15)	Radiation	404	RC	4.4	-	-	-	99 (25)	-	195 (48)	-	-	-
Martinez et al. (8)	Radiation	26	RC	3.9	10 (38)	-	3 (11.5)	7 (27)	19 (73)	16 (61)	9 (35)	-	4 (40)
Gontero et al. (33)	Radiation	209	RC	2.4	41 (20)	228	71 (34)	-	-	-	134 (64)	-	8 (17)
Ogaya-Pinies et al. (31)	Mixed (majority radiation)	96	R	1.2	85 (88.5)	125	25 (26)	16 (17)	-	15 (16)	55 (57)	-	17 (18)
Lama et al. (29)	Mixed (majority radiation)	78	R	10.8	-	-	39 (50)	23 (29)	-	11/57 (19)	-	33/62 (53)	1/16 (6)

**Table 2:** sRARP after ablation therapy failure. Studies in the narrative review which reported outcomes specifically for ablation therapy failure are included. Study type is reported as either retrospective (R) or retrospective comparative (RC).

Study	Therapy	N	Nerve sparing (unilateral or bilateral), n (%)	Study type	Median/mean follow-up time (years)	Median/mean operative time (minutes)	Postoperative complications, n (%)	Positive surgical margin, n (%)	Upstaging on sRARP specimen, n (%)	BCR, n (%)	Continence (1-year), n (%)	Continence (>1 year), n (%)	Potency
Marconi et al. (52)	Not specified	82	62 (76)	R	-	-	5 (6)	11 (13)	-	34 (41)	64/77 (83)	-	14/72 (14)
Nunes-Silva et al. (64)	Not specified	22	20 (91)	RC	-	135	2 (9)	-	-	-	7/13 (54)	-	-
Nathan et al. (9)	Mixed whole gland	49	5 (10)	RC	1.4	160	11 (22)	17 (35)	20 (41)	18 (37)	23 (49)	26 (53)	1 (2)
	Mixed focal	86	31 (36)			165	7 (8)	34 (40)	40 (47)	13 (15)	63 (78)	72 (84)	6 (7)
Espinós et al. (54)	Cryotherapy	12	-	RC	2	154	3 (25)	0 (0)	-	6 (50)	3/4 (75)	8/11 (73)	3/4 (75)
							
	HIFU	6	-				1 (17)	1 (17)	-	1 (17)	3 (50)	-	1/1 (100)
Spitznagel et al. (58)	HIFU	13	13 (100)	R	-	260	6 (46)	1 (8)	3 (23)	0 (0)	-	-	-
Thompson et al. (59)	HIFU	45	13 (29)	R	1.5	140	18 (18)	20 (44)	24 (53)	4/38 (10.5)	27/42 (65.5)	-	0 (0)
De Luca et al.	HIFU	11	3 (27)	R	1	110	1 (9)	3 (27)	-	1 (9)	9 (81)	-	2 (18)
De Groote et al. (53)	Mixed	106	3 (3)	RC	2.1	142	-	42 (39)	44 (41.5)	14 (13)	32/103 (31)	20/40 (50)	5 (5)
	HIFU alone	59	-			-	-	23 (39)	-	11 (19)	-	-	-
	Cryotherapy	1	-			-	-	0 (0)	0 (0)	0 (0)	-	-	-

Due to improved visualization, magnification, and improved tissue control, the minimally invasive approach to sRARP may result in improved oncologic outcomes. The feasibility of the sRARP has been demonstrated. However, a majority of studies suffer from a lack of long-term follow-up and thus poor, if any reported survival parameters. Some comparative studies do exist. When comparing open to robotic approaches to salvage prostatectomy, Martinez et al. found no differences in 2-year BCR free survival (67% vs 60.9%, p=0.873) with an overall CSS of 95% in their study of 76 patients (8). Grubmueller et al. found no differences regarding PSM rates in the open vs laparoscopic/robotic approaches (26.8% vs 21.8%, p=0.13) or final pathological staging however did note that rate of node positive disease was higher in the minimally invasive approaches (28). Yuh et al. found a BCR free survival of 57% at 3 years and a CSS of 100% at 5 years. On multivariate analysis, they found that PSA level prior to sRARP and ECE on final pathology were predictors of recurrence or disease progression (29). Kaffenberger et al. found that both pre-radiation Gleason grade and PSA doubling time were associated with decreased BCR-free survival though margin status was not, in this study (30). In an effort to standardize patient selection for sRARP, the EUA determined that ideal candidates should have PSA <10 ng/mL, Gleason grade ≤3, no obvious lymphadenopathy, and clinical stage ≤T2. A validation study on these guidelines by Calleris et al. demonstrated a better MFS (90% vs 76%, p=<0.001) and OS (89% vs 84%, p=0.01) at 5 years in patients meeting the EUA guideline criteria (11). Ultimately, sRARP for post-radiotherapy BCR prostate cancer provides acceptable oncologic outcomes which are worse when compared to pRARP. sRARP performed for those with favorable BCR as outlined by the EUA guidelines provide more favorable outcomes than those with radio-recurrent prostate cancer and concomitant high PSA values and worse pathologic stage.

#### c.Complications of sRARP after Radiotherapy

Traditionally, salvage prostatectomy was avoided and often vilified due to high rates of complications ranging from rectal injuries to urinary leaks. Initially, reported rates of rectal injury were as high as 20% (15). As such, only 3% of patients will receive sRARP (31). More recent studies have demonstrated this complication to occur in fewer than 2% of patients, partly due to the use of robot-assistance (11, 32, 33). The most frequent complication in these series is bladder neck contractures ranging from 14.7-50%. Recently, in a multi-institutional retrospective study of 295 patients undergoing salvage radical prostatectomy using either the open (186) or robotic (209) approach, Gontero et. al. reported that the robotic approach was associated with a shorter hospital stay and lower rate of urethral stricture (15.8% vs 7.7%) and in a multivariate analysis, the robotic approach was the only independent predictive factor for preservation of urinary continence (34). Other commonly reported complications include vesicourethral anastomotic leaks, rectourethral fistula, enterotomies, pulmonary emboli and sepsis however, none of the aforementioned complications occurred in more than 4% of patients (35). In a meta-analysis comparing sRARP to pRARP, Zargar et. al. found a lower rate of complications requiring re-intervention in pRARP compared to sRARP (3% vs 14%). This highlights the increased complication rate associated with sRARP that should not be discounted.

In recent years, the Retzius sparing approach has been utilized in the salvage setting to minimize the risk of certain complications, namely rectal injuries. By directly visualizing the rectum during posterior dissection, the plane can be developed with more certainty. This approach was initially described in 2013 and the outcomes were reported in ensuing years (36-38). Compared to standard approach, Retzius sparing sRARP was associated with a lower 30-day complication rate (10% vs 26%) though this did not significantly differ. Only a single rectal injury was noted in the standard approach and one intraoperative complication was noted which was a ureteral injury in the Retzius sparing approach requiring ureteroneocystostomy (39). Similarly, Kowalczyk et al. found a similar complication rate between standard and Retzius sparing sRARP in a study of 72 patients. (40). Ultimately, the use of robot-assistance has drastically decreased complication rates and the Retzius sparing approach may further reduce rectal injury rates, though larger studies are needed to make definitive conclusions.

#### d.Functional outcomes of sRARP after Radiotherapy

In comparison to the open approach, sRARP has been associated with improved functional outcomes. In a study of 395 patients undergoing open or robotic salvage prostatectomy, an overall 75.4% continence rate was found with the robotic approach being an independent predictor of continence (34). In a comparison of patients undergoing pRARP and sRARP, Bates et al. found lower rates of continence (76.9% vs 96.2%) and potency (31.5% vs 49%) for sRARP compared to pRARP. In those patients with a return of continence, a longer time to continence was noted in those undergoing sRARP. Furthermore, in this study only 7.6% of patients underwent bilateral nerve sparing in the sRARP arm compared to 34% in the pRARP arm though no differences in functional outcomes were noted between these groups (41). Yuh et al. reported a continence rate of 45% at 6 months and a return of erectile function of 23% in those that had good erectile function prior to sRARP (29). Ogaya-Pinies et al. found a continence rate of 57.3% at 12 months and a potency rate of 55% in those undergoing sRARP (31). Retzius sparing sRARP has been reported to improve continence rates compared to traditional sRARP because of improved sphincter visualization. Retzius sparing sRARP has been associated with improved continence compared to traditional sRARP (78.4% vs 43.8%) and a quicker return to continence (47 vs 180 days) (40). It becomes clear that sRARP has improved continence outcomes compared to open salvage prostatectomy however compared to pRARP, patients undergoing sRARP have worse functional outcomes. Though, the Retzius sparing approach improves continence rates compared to standard sRARP, comparative studies or pRARP to Retzius sparing sRARP may provide more definitive information.

## sRARP after Ablative Therapies

### a.Outcomes of Ablative Therapies

Focal and whole gland ablative therapies are minimally invasive treatment modalities for localized prostate cancer. The most utilized modalities include cryotherapy, high-intensity focused ultrasound (HIFU), transurethral ultrasound ablation (TULSA), irreversible electroporation (IRE), focal laser ablation (FLA). Central to the concept of ablative therapy is the treatment of prostate cancer while minimizing the comorbidities of extirpative surgery (42). While becoming integral in the armamentarium of localized prostate cancer management, post-ablative surveillance remains challenging. The diagnosis of BCR after ablation has its basis in PSA recurrences as no individualized definition has been proposed. The ASTRO criteria are most often used to determine BCR after prostate ablation, though this is likely an imperfect measure of oncologic viability. Acknowledging the limitations therein, several studies have examined BCR rates at intermediate-term follow-up. The 5-year, post-cryotherapy BCR rates range from 4-87% (42–47). For HIFU, 5-year BCR rates range from 50-65% (48, 49). For TULSA, BCR rates have ranged from 3-27% (50, 51). The large range in reported rates of BCR is likely due to the lack of pathologic homogeneity and stratified survival analyses in these studies. IRE involves electrical impulses to irreversibly increase cell permeability, reducing cell homeostasis capability. A large study of patients undergoing focal IRE demonstrated a 3-year cancer control rate of 77.5% and when allowing for one re-treatment, only 4.8% of patients required subsequent whole-gland treatment. More detailed outcomes of whole gland and focal therapies are readily available (52). Regardless, BCR after ablative therapy warrants additional management and sRARP remains a frequently performed treatment modality in this group. Thus, it is critical to understand the rates of PSM, BCR, upstaging and the impact of these factors on survival outcomes in this setting.

### b.Oncologic Outcomes of post-ablative sRARP

Most of the available literature regarding post-ablative sRARP are not segregated by ablative modality. Several larger studies have been published and dissection of these results may provide some insight. One of the largest series of post-ablative sRARP demonstrated a 13% rate of PSM at time of sRARP and ultimately a 42% BCR rate at 2-year follow-up. Progression-free survival in this cohort was 73.9%, 48%, and 36.2% at 1, 2, and 3 years, respectively. In this study, both pathologic stage T3b and PSM were identified as independent risk factors for BCR (53). A study by De Groote et al. incorporated multiple ablative modalities and demonstrated an overall PSM rate of 39% (54). BCR rate was 19% in patients having received prior HIFU and no BCR noted in either cryoablation or IRE. Additionally, there were no differences in PSM, BCR rates, or recurrence-free survival in patients receiving either whole gland or focal ablation. Total upstaging rate in this group was 33% at the time of sRARP though the consequences of this are not certain. Of note, this study incorporated multiple ablative and radiation modalities, thus care must be taken when interpreting the relevance to sRARP for post-ablative BCR, alone (54). Similarly, Nathan et al. demonstrated that regardless of whether patients received prior focal or whole gland ablation, rates of PSM (39.6% vs 34.7%) and BCR (29.1% vs 36.7%) were similar at median follow-up of 1.5 and 1.2 years, respectively (9). Conversely, Linares Espinos et al. found a 14% PSM rate in a study of 28 patients undergoing sRARP or laparoscopic radical prostatectomy, after ablative therapy, with a BCR rate of 39% with a time to BCR of 16 months (55). Operative times and rates of upstaging in this study was higher in the whole gland group compared to focal ablation group (70% vs 28%). A study by Herrera-Caceres et al. of 34 patients undergoing both open and robotic salvage prostatectomy demonstrated a BCR rate of 20.6% at 4.3 years and as expected PSM was associated with worse BCR-free survival (32). Amongst ablative therapies, IRE is possibly the most contemporary technique. When examining post-IRE sRARP, van Riel et al. found a 25% rate of PSM and only a 2.5% rate of BCR, though 1 patient never achieved an undetectable PSA and 1 patient developed metastatic disease (56). Though some studies have demonstrated equivalent oncologic outcomes, time to BCR in patients undergoing post-ablative sRARP are inferior. PSM and BCR rates for post-ablative sRARP range from 13-39% and 0-42%, respectively. This is compared to 26% and up to 36% for pRARP in high-risk patients, at 10 years (57, 58). Ultimately, very few studies exist examining oncologic outcomes of a single ablative therapy and the majority of studies have small samples sizes. Furthermore, the accuracy of Gleason scoring in patients having undergone ablation is not certain and as such definitive conclusions regarding the persistence of malignancy are difficult to make. Finally, follow-up in post-ablative sRARP cohorts is relatively short compared to the natural disease course of prostate cancer and thus longer-term follow-up is needed to determine the implications of post-ablative sRARP.

### c.Complications after post-ablative sRARP

Complications for sRARP after failed ablative therapies have generally been similar to pRARP. Spitznagel et al. reported a series of 13 patients undergoing sRALP after HIFU. Compared to patients undergoing pRARP, a similar rate of complications 46% was noted in a sRALP cohort (59). Similarly, post-ablative sRARP complication rates have ranged from 39% to 44% in other studies (9, 54). In a study of 45 patients undergoing sRARP, Thompson et al. reported a 90-day complication rate of 18% which was similar to the complication rate of their previously pRARP series (60–62). In a study of 11 patients undergoing post-HIFU sRARP, De Luca et al. found only a single post-operative minor complication (63). Peretsman et al. reported no complications in patients undergoing post-HIFU sRARP (64). Linares Espinós et al. published on a mixed ablative therapy cohort with 6 patients who failed HIFU and underwent sRALP, of which 1 patient had a postoperative complication (55). van Riel et al. found a 0% major complication rate in 39 patients undergoing sRARP after IRE, though minor complication rate was not reported (56). In a study of 82 patients undergoing sRARP after any ablative therapy, a 4.8% minor and 1% major post-operative complication rate was reported (53). In summary, intraoperative complications in post-ablative sRARP are negligible, and postoperative complication rates are comparable to pRARP (53, 65–67). While pRARP is widely performed, a majority of sRARP are likely performed at tertiary centers and thus, while feasible and safe, should be performed by experienced surgeons.

### d.Functional outcomes after post-ablative sRARP

Both erectile function and continence status are important measures of patient satisfaction after surgery for prostate cancer (68). Traditionally, sRARP was thought to be associated with worse functional outcomes compared to pRARP. Prior work on continence recovery after pRARP has indicated that younger age at surgery and less intraoperative blood loss may be favorable predictors of continence recovery (69), but results after sRARP are less clear. Definitions of continence ranges from no pad usage to questionnaire-based determinations and potency definitions range from non-assisted erectile function to the allowance of oral phosphodiesterase inhibitors and vacuum erection devices. Continence rates for post-ablative sRARP at 1 year post-operatively range from 31-92% with higher rates in those having undergone focal compared to whole gland ablation though no correlation is evident based on continence metric used (9, 53–55, 59, 63, 65). Related to potency, Marconi et.al found a 14% potency rate, De Groote et al. reported a 5% potency rate, Thompson et al. reported a 0% potency rate, and Nathan et al. found a 2% potency rate for whole gland and 7% for focal ablation (53, 54, 60, 62). Similarly, Nunes-Silva et al. found significantly lower IIEF-5 scores in post-ablative sRARP patients compared to those undergoing primary RARP (65). Further confounding factors to making conclusions is the general lack of pre-ablation and pre-sRARP erectile function data in these patients, however Espinos did find that among the 4 patients that were potent prior to sRARP, 75% retained erectile function, post-operatively (55). Nerve-sparing attempts in this setting have been attempted but ultimately, oncologic control should not be sacrificed. Spitznagel performed nerve sparing in all 13 of their patients undergoing post-HIFU sRARP and found similar erectile function recovery compared to patients undergoing pRARP (59). Others were only able to successfully perform unilateral nerve sparing in 3% of patients and only 1% this group were able to maintain pre-operative potency (54). This is in contrast to pRARP in which potency rates approach 54-97.4% depending on unilateral versus bilateral nerve sparing (57). While many studies investigating post-ablative salvage prostatectomy are confounded by combinations of focal and whole gland ablation, pure laparoscopic and robotic surgical approaches, and variability in defining potency and continence, it is clear that patients undergoing sRARP have worse functional outcomes compared to primary RARP.

## SURGICAL DESCRIPTION, TIPS, AND TRICKS

Prior to performing sRARP it is imperative to be familiar with the available robotic platform. The surgeon and staff should have knowledge and experience of the daVinci system controls and all equipment should be prepared prior to case commencement. In order to optimize patient outcomes and minimize complications, patient selection is imperative.

### Patient positioning, robot docking, and initial steps

**Figure 1:**
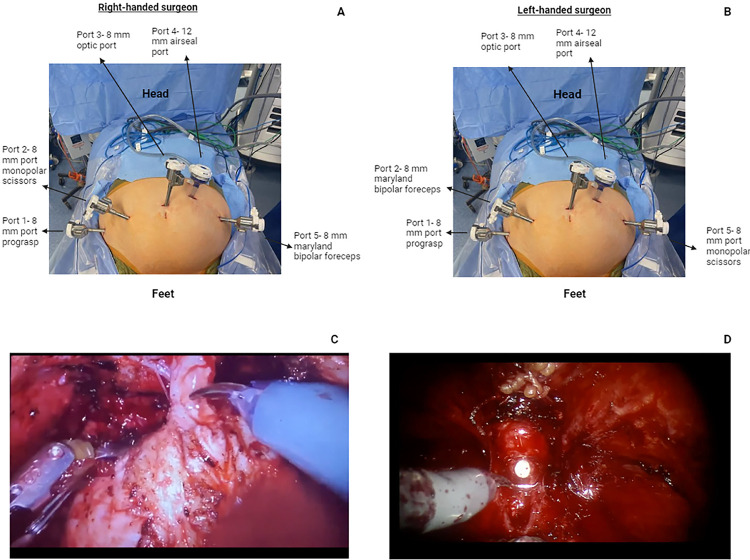
sRARP port placement and instruments with intraoperative Imaging.

We place the patient in low lithotomy position and secure them with shoulder bolsters. Arms are secured to the patient’s side and legs are placed in yellow fin stirrups. A tilt test is performed. After sterile preparation with chlorhexidine scrub and draping, a stab incision is made superior to the umbilicus. A Veress needle is used to obtain access to the abdomen, cephalad to the umbilicus, and a water drop test is performed. The abdomen is then insufflated to 15mmHg using CO2. The needle is removed, the incision is expanded laterally and a 8mm blunt or bladed trocar is placed through this incision. Bariatric trocars may be used if needed. The patient is placed in steep Trendelenburg position. Additional trocars are placed according to Figure-1 and the robot is docked between the patient’s legs. Instrument placement depends on surgeon handedness and is described in Figure-1.

(A) Right-handed surgeon setup. (B) Left-handed surgeon setup. The robot is docked in between the patient’s legs as pictured. (C) Urethra during sRARP after post-proton beam radiation therapy failure. (D) Hypervascular urethra during sRARP after external beam radiation therapy.

### Initial dissection, dorsal venous complex, and management of endopelvic fascia

While a posterior approach may be used, we favor an anterior approach using the midline trocar as the optic port. The anterior peritoneum is incised in between the right and left vasa deferentia, dividing the median and medial umbilical ligaments. This dissection is carried distally in the avascular retropubic space until the bilateral endopelvic fascia and superficial dorsal venous complex (SVC) are encountered. There is typically very little desmoplastic reaction in this space. The endopelvic fascia however, may be severely scarred requiring cautery followed by blunt dissection. During this step, the fourth arm should be used to lateralize the prostate to provide adequate exposure, contralateral to the working side. The SVC may be fulgurated and divided. We recommend controlling the deep dorsal venous complex (DVC) with suture ligation in a figure of eight fashion using either a monofilament or braided suture on a CTX needle.

### Anterior and posterior bladder neck

The fourth arm is then used to gently pull the bladder cephalad. The Foley catheter is pulled to ascertain the location of the anterior bladder neck. Monopolar scissors are used to incise the anterior bladder neck. This is carried down posteriorly until the bladder neck is opened. We recommend a wide bladder neck to ensure negative margins. The Foley catheter is deflated, grasped, and pulled upwards to anterior abdominal wall using the fourth arm. The bedside assistant is then asked to retract the bladder cephalad with the suction tip to allow access to the posterior bladder neck and the ureteral orifices. Dissection is carried down through the posterior bladder neck and the suction tip is advanced to provide continuous retraction until the vas deferens is identified. The posterior dissection is often more difficult in the salvage setting due to fibrosis. A similar posterior bladder dissection technique has been described for RARP after Urolift® (70).

### Posterior dissection, vascular pedicles, and vesicourethral anastomosis

The vas deferens is grasped and retracted anteriorly and cephalad. Using spot cautery and a posterior sweeping motion, the vas deferens is dissected free and divided distally. The fourth arm is used to grasp the vas deferens to the contralateral side and the SV is dissected free in a similar fashion. This is repeated on the contralateral side. The dissection plane is carried out more posteriorly in the salvage setting to ensure negative margins. The posterior dissection is carried out in the midline to the level of the prostatic apex and then performed medial to lateral coming back to the bladder neck.

In the salvage setting, negative margins are of utmost importance and as such, we use the extra-fascial approach. The prostate is placed on traction with the first arm to ensure the rectum does not tent upwards. At this point, a vessel sealer is used to divide the prostatic pedicles close to the prostate from base to apex. Conversely, medium hem-o-lock clips can be used to secure prostatic pedicles. This is repeated on the contralateral side. When dividing the apex, the first arm is used to traction the prostate downwards and the monopolar scissors are used to divide the DVC. Once through the DVC, the fourth arm is used to traction the prostate cephalad to allow apical dissection behind the DVC. Monopolar scissors are then used to divide the anterior prostate apex until the Foley catheter is visualized. When dividing the posterior apex, it is critical to place the Maryland forceps underneath this layer to prevent rectal injury, particularly in the salvage setting where the posterior urethra may be fibrous and adherent to the rectum. A similar technique has been described to repair stenosis of failed vesicourethral anastomosis in primary open prostatectomy (71). If Denonvillier’s fascia is robust, we use a 3-0 V-lock, barbed suture to perform a Rocco stitch. Bladder neck reconstruction is often required using 2-0 PDS cut to 6 inches, at the 3 and 9 o’clock position. The vesicourethral anastomosis using a 2-0 barbed suture in a circumferential, running fashion over a 20Fr Foley catheter starting outside to inside on the bladder, and inside to outside on the urethral stump.

If there is concern for a rectal injury, the pelvis should be filled with saline, and air should be instilled in the rectum via a rectal probe or catheter. If air bubbles are noted, there is likely a rectal injury and consultation with general surgery should be considered for colostomy. In the absence of significant contamination, a two layered repair may be considered.

### Post-operative management

The immediate post-operative management of these patients involves a 23 hour stay with drain removal prior to discharge if output remains <200cc/24 hours. Drain creatinine testing is optional depending on the drain output. Foley catheter duration is typically 10 days if the urethra appears well-vascularized and nonfriable. Cystograms are not routinely performed. However, if the urethra is friable and pale with apparent poor vascularity (Figure-1c), the urethral catheter is left for 14 days with a cystogram prior to catheter removal.

## CONCLUSIONS

Salvage robot-assisted laparoscopic radical prostatectomy is the surgical treatment of choice in biochemically recurrent prostate cancer after radiation or ablative therapies. Complication rates were high and functional outcomes were poor in the era of open prostatectomy and in the early days of robotic surgery. Technical refinement, improved anatomic understanding and robotic capabilities have reduced post-operative complication rates. Oncologic measures remain worse than in those patients undergoing primary surgery however when salvage surgery is offered to those patients with low-risk BCR, oncologic outcomes are improved compared to those with high-risk BCR. Potency and continence rates in the salvage setting are poor, though continence is somewhat improved using the Retzius sparing approach. Admittedly, head-to-head studies and randomized controlled trials comparing sRARP after various radiation and ablative therapies, would be difficult to perform. However, retrospective cohort studies directly comparing outcomes between various radiation and ablative therapies merit further investigation.
